# Magic for the mind's eye: A promising avenue for more universal design in the art of magic

**DOI:** 10.1177/20416695231222995

**Published:** 2024-01-08

**Authors:** Vebjørn Ekroll, Lara Wünsch, Rob van Lier

**Affiliations:** Department of Psychosocial Science, 1658 University of Bergen, Bergen, Norway; Donders Institute for Brain, Cognition and Behaviour, Radboud University, Nijmegen, The Netherlands

**Keywords:** magic, imagery, blindness, universal design, inclusion

## Abstract

In the art of conjuring, as well as in cognitive science, possibilities for designing magic tricks that are suitable for people who are blind or visually impaired have only rarely been considered. In this article, we argue that many magic tricks which are normally presented in a visual way, are not inherently based on visual processes, but instead on systematic illusions and limitations in visual imagery and mental simulations. Accordingly, it should be possible to adapt these tricks for presentation in a non-visual format, which should be suitable for people who are blind or visually impaired. As an initial test of this general approach, we adapted three magic tricks for non-visual presentation and presented them for blindfolded participants. Standard versions of the tricks were also presented to seeing participants. The participants in both groups were asked to indicate how magical they felt the tricks were, as well as whether they had any idea about the secrets behind them. The results suggest that the non-visual versions of the tricks are roughly comparable to the regular visual versions. We conclude that adapting magic tricks based on illusions of imagery for non-visual presentation appears to be a promising avenue for more universal design in the art of magic. We also argue that the illusions of imagery responsible for the experiences of magic evoked presents interesting challenges for basic cognitive science.

There were 45 minutes of show, where a large number of children laughed, enjoyed and applauded my magic effects, while **MARA** was only told by her teacher. I felt really bad and thought, if one person couldn't enjoy my show, it was useless. So I said to myself, **I have to do magic for the blind**.

— Willy Tidona (2017)

Is it possible to develop and perform magic tricks that are equally interesting and enjoyable for people who are blind or visually impaired as for anybody else? And if so, how? These questions are important because the art of conjuring, being a cultural activity that both entertains and educates, ideally should (in line with the UN Convention on the Rights of Persons with Disabilities, particularly §24 and §30) be made available to people with disability in accessible formats. Furthermore, these questions are also interesting from a purely theoretical point of view because understanding the mechanisms involved in the creation of magical experiences poses an interesting challenge for basic cognitive science.

Given that major parts of entertainment magic are strongly associated with visual effects and visual illusions, one might be prone to think that performing magic tricks for people who are blind would be a futile exercise. It is probably indeed true that some tricks are inherently limited to visual presentation to seeing spectators because they rely on visual illusions which are by-products of the normal functioning of the visual system, but in the case of many other tricks, the association with the sense of vision may be due to mere biases in the historical development of the art. Most magicians^1^ and most of their *attenders*^2^ see—simply because most people do—and this may have biased the development of the art and its repertoire towards tricks, or ways of presenting them, which are particularly convenient or suitable for an audience of seeing attenders.

In light of recent psychological theories, according to which the essential feature of an experience of magic is a cognitive conflict between our immediate experiences and what we believe to be possible (Kuhn, 2019; Leddington, 2016), however, there is no a priori reason to attribute any special role to the sense of vision over and above other senses or other parts of our mental apparatus. Surely, the sense of vision is only one among many other faculties that can be exploited for creating immediate experiences that are in conflict with what people intuitively believe to be possible.

It appears rather self-evident that the genre of magic known as *mentalism* or *mental magic* includes many effects and tricks which are suitable for non-visual presentation. The Chilean magician Juan Esteban Varela is known for his magic show “From the Dark,” where both the magician and the audience are blindfolded. Unfortunately, we have not had the chance to attend this show, but judging from his DVD titled “Zero elements” (Varela, n.d.), we presume that he is mainly using various forms of *conjuring with information*^3^, such as apparently predicting people's choices or reading their minds. Thus, we can be fairly confident in stating that some magic tricks are suitable for non-visual presentation, while others are not. The interesting open question, though, is whether our notions about what kind of tricks or magic effects are suitable for non-visual presentation are realistic and to what extent and by what means the potential for non-visual presentation of interesting magic tricks can be maximized. The Argentinian magician Willy Tidona (2017) has published an account of his efforts directed towards making his art accessible and enjoyable for people who are blind or visually impaired, which contains several concrete examples illustrating how this can be achieved. Magic tricks developed to be performed over the radio may also be of interest. For instance, it appears plausible that the majority of the tricks described by Tamariz and Navarro (2008) are suitable for adaptation to the requirements of people who are blind or visually impaired because they are based on conjuring with information and mathematical magic. Most of the tricks would need to be suitably modified, though, because they require the listeners to manipulate and look at visual materials such as regular cards. One of the tricks described by Tamariz and Navarro (2008), however, called “The multiplication of Bread” (p. 113), is likely to be effective without any modification, because it relies on a purely tactile illusion known as Aristotle's illusion (Hayward, 2008). This illusion can be experienced by crossing the index and the middle finger and touching a small object (such as a pea or the tip of your own nose) placed between the crossed fingertips while having your eyes closed. Typically, one will then have the experience of touching two objects rather than just one.

In the present work, we focus on a seemingly promising avenue for adapting magic tricks for non-visual presentation inspired by Ekroll's (2019) notion of “topological tricks” based on “illusions of imagery.” The central idea is that a large set of magic tricks, which can be loosely described as topological tricks (Gardner, 1956) since they involve flexible materials such as paper, cloth, rope, and rubber bands are highly robust because they are based on systematic illusions and limitations in visual imagery and mental simulations. Importantly, if the notion that the essential factors responsible for the magical effects evoked by these tricks are illusions of imagery rather than visual illusions, these tricks should be suitable for non-visual presentation.

As an initial test of this general approach, we compared three magic tricks presumptively based on illusions of imagery presented in their original visual format with corresponding adapted versions designed to be presentable in a non-visual format to blind or blindfolded participants. Our hypothesis was that the non-visual versions of these tricks are about as effective as the original visual versions. To anticipate, the results suggest that this is indeed the case.

## Methods

The three magic tricks of central interest in this experiment were

- a variant of the *wholesale ring removal* trick described by James (1975, p. 76), which we shall refer to as the *ring to cup trick*,
- Robert Neale's *trapdoor card* (Fulves, 1985; Harris, 2019) and
- one of Gardner's (1978) variants of the *linking paperclips trick* invented by Bill Bowman (Butler et al., n. d.; Ganson, 1994, p. 103).

These tricks, which we henceforth refer to as the *imagery tricks*, were selected as examples of tricks we believe to be based on illusions of imagery and hence readily adaptable for non-visual presentation. One group of participants (the *seeing group*) experienced standard versions of these tricks presented visually, while a second group of participants (the *blindfolded group*) experienced adapted versions of these tricks while wearing a blindfold. In addition to these three imagery tricks, we also presented a prototypical *visual trick* and a prototypical *mental magic trick* to each participant. These two tricks were presented in the same standard (non-blindfolded) version to both groups of participants. The purpose of including these tricks was to obtain an indication of how well the imagery tricks work compared to other typical magic tricks. The visual trick was a version of the cut and restored rope trick (Barnhart, 2010), which we have good reason to believe is primarily based on the visual illusion of amodal completion (Ekroll et al., 2017; 2018). The mental magic trick was the classic *grey elephant in Denmark trick* (Clark, 2012; Tidona, 2017) made famous by and sometimes attributed to Max Maven.

Table 1 gives an overview of the tricks presented to the participants in the two groups. As can be seen, only the version of the imagery tricks was different for the two groups. The blindfolded group wore blacked-out swimming googles which made it impossible to see during the performances of the imagery tricks.

**Table 1.** Overview of the tricks presented to the two groups of participants.

Group	Visual trick	Mental magic trick	Imagery tricks		
Blindfolded group	Cut and restored rope	Grey elephants in Denmark	Ring to cup, *non-visual* version	Trapdoor card, *non-visual* version	Linking paperclips, *non-visual* version
Seeing group	Cut and restored rope	Grey elephants in Denmark	Ring to cup, *visual* version	Trapdoor card, *visual* version	Linking paperclips, *visual* version

In the seeing group, visual versions of the imagery tricks are used, and in the blindfolded group, non-visual versions of the imagery tricks were used. Otherwise, the same tricks were used in the two groups. That is, the visual trick and the mental magic trick were presented without a blindfold in both groups. The tricks are described and explained further below in the main text.

The tricks were performed live by the experimenter (author LW) and presented in random order to each of the participants in individual sessions.

We recruited 60 participants by approaching people in the cafeteria areas at the University of Bergen and every other participant was assigned to each of the two groups (seeing or blindfolded). After running the first eight participants from each group, the experimenter noticed a potential problem with the presentation of the imagery tricks in the blindfolded condition, namely that she did not hide the props before the participant were allowed to remove the blindfolded, meaning that they could use the sense of vision to at least see the end result of the magic trick (say, the cup being tied to the rope in the ring to cup trick). After realizing this, we decided to make sure that the participants in the blindfolded condition were never allowed to see any of the props used in the imagery tricks. The first eight participants in the blindfolded group were therefore excluded from analysis, meaning that we ended up with 30 valid data sets in the seeing group and 22 in the blindfolded group.

After providing informed consent, the participants were asked to state their approximate age (response alternatives: 18–21, 22–25, 26–29, 30–34, 35–39, or 40 or above) and gender (response alternatives: Female, Male, Non-binary, or Other). Then, the experimenter proceeded to perform the five magic tricks in the predefined random order (see above). After each trick, using a response form on a laptop, the participants were asked to indicate “How magical was this trick?” on a 0 (not magical at all) to 10 (extremely magical) scale. They were then asked “Do you have any idea what the secret behind this trick might be?” and then “If Yes, please describe your idea in the box below.” At the end of each individual session, the participant was asked whether they had any idea about the aim of the experiment. This was done in order to check for potential demand characteristics, as suggested by Orne (1962). None of the participants provided an answer indicating that they had correctly guessed the research hypothesis. The participants where then debriefed by offering them the opportunity to ask any questions they might have about the experiment or the magic tricks. Each session lasted about 12 min in total.

Table 2 shows the distributions of gender in the two groups. The distributions are very similar in both groups. Table 3 shows the distribution of age range in the two groups. Again, the distributions are very similar in both groups.

**Table 2.** Distribution of Gender in the Two Experimental Groups.

		Gender		
		Female	Male	Total
Group	Blindfolded	13 (59.1%)	9 (40.9%)	22 (100%)
	Seeing	18 (60%)	12 (40%)	30 (100%)
	Total	31 (59.6%)	21 (40.4%)	52 (100%)

**Table 3.** Distribution of Age Range in the Two Experimental Groups.

		Age range			
		18–21	22–25	26–29	Total
Group	Blindfolded	14 (63.6%)	7 (31.8%)	1 (4.5%)	22 (100%)
	Seeing	19 (63.3%)	10 (33.3%)	1 (3.3%)	30 (100%)
	Total	33 (63.5%)	17 (32.7%)	2 (3.8%)	52 (100%)

The tricks used in the experiment are described in the following paragraphs.

### The Cut and Restored Rope Trick

In this classic trick, the magician appears to cut a rope in the middle with a pair of scissors, but swiftly reveals that the rope is unsevered. A performance of the trick, which is described in Barnhart (2010) and in James (1975, p. 135), can be seen in Movie 1. The secret handling of the rope is shown on p. 135 in James (1975). Essentially, the secret behind the trick is that although the rope appears to be cut in the middle, only a small part of one end is cut off. Amodal completion (van Lier & Gerbino, 2015) based on the Gestalt principle of good continuation is arguably a central psychological factor contributing to the effectiveness of this trick (Ekroll et al., 2017; 2018).

### The Grey Elephants in Denmark Trick

In this trick, the attender is asked to “think of a small number, perhaps something under 10 to make doing the mental maths easier” (Clark, 2012). The participant is then asked to do a few simple calculations starting with this number, convert the resulting number into a letter (at the corresponding position in the alphabet) and think of a country starting with this letter, “Nothing crazy … just the most logical and common one that comes to mind” (Clark, 2012). The attender is then asked to think of an animal starting with the second letter in name of the country they are thinking of, as well as the color of that animal. The magician then reveals that they know that the attender is thinking of a grey elephant in Denmark, by saying “This is pretty strange, the last time I was in Denmark, there were no grey elephants anywhere.” The secret behind the trick is that the calculations the attender is asked to perform in the beginning always lead to the number 4, which corresponds to the letter D. Furthermore, most people will think of Denmark^4^, where the second letter is ‘e’, and based on that, most people will think of an Elephant. The computations that the attender is asked to perform at the beginning is to double the number they are thinking of, add 8 to the result, divide the total so far by 2 and then subtract the number they were thinking about at the start from it. Written up in algebraic form, where X is the number the attender was thinking of, it is straightforward that the result is always (2X + 8)/2−X = X + 4−X = 4 irrespective of what number the attender was thinking of.

### The Ring to Cup Trick, Visual Version

**Figure 1.**
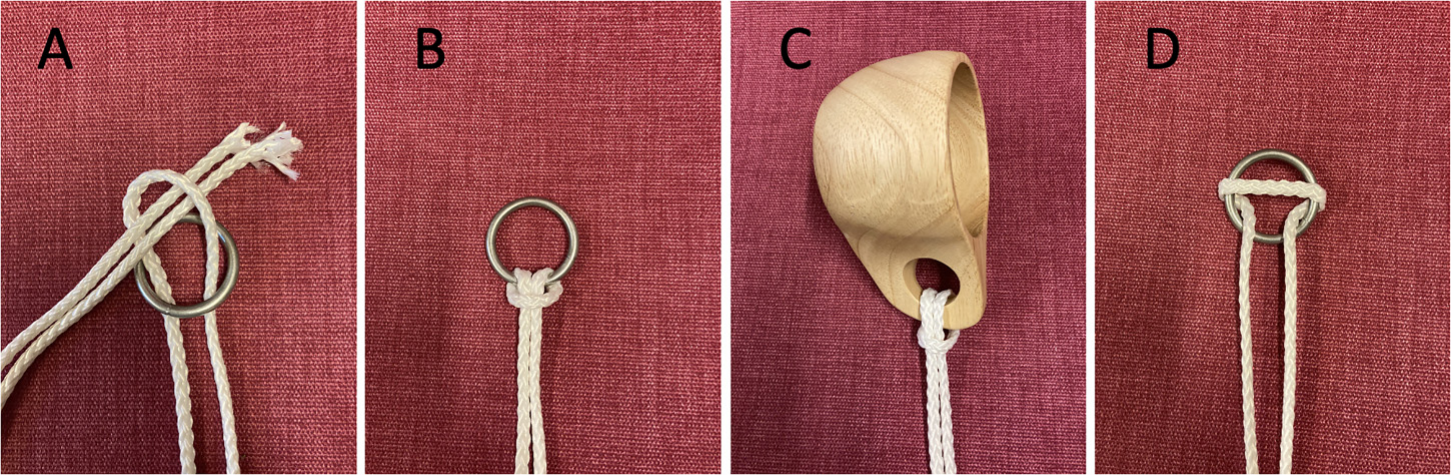
The ring to cup trick. (A) The lark's head knot in (B) is first tied by folding the rope in the middle, threading the resulting loop through the ring, threading the ends of the rope through the loop and pulling the rope taut. (C) During the trick, the ring is replaced by a cup. (D) Illustration of how the ring in (B) can easily be released from the rope although the attender holds on to the ends of the rope.

In the ring to cup trick (see Movie 2), the attender is first handed a rope and a metal ring and asked to check that “everything was ok with them.” They were then instructed to tie the ring to the rope by first folding a rope in the middle to form a loop which is threaded through the ring, and then threading the two ends of the rope through the loop (Figure 1A). By pulling the two ends tight through the loop, the lark's head knot shown in Figure 1B results. Instructions are given verbally, with the aid of manual gestures. The instructions are adapted on the fly in case the participant failed to tie the knot as intended. Once the attender had completed the knot as intended, the magician points out to the attender that the ring is now securely tied to the rope and asks the attender to hold on to the two ends. While the attender holds on to the two ends, the magician puts the end with the ring into an opaque shopping bag. After a short while, the attender is asked to retrieve the ring by pulling the rope back towards him-/herself. As if by magic, the ring is now replaced with a cup (Figure 1C). The secret behind the trick is that the ring can easily be untied from the rope by “wrapping” it over the ring as shown in Figure 1D and Movie 3. By reversing this “unwrapping” the cup is easily tied onto the loop. It is currently not known why people tend to have difficulty realizing that the knot can be undone as shown in Figure 1D, but Ekroll (2019) speculate that it may be because the knot “is mentally represented in terms of the actions made to tie it.” Hence, since untying the knot by reversing the sequence of actions used to tie it is impossible (remember that the attender is asked to hold the two outer ends of the rope), the solution is not available in the attender‘s mental representation of the situation.

### The Ring to Cup Trick, Non-visual Version

The non-visual version of this trick was identical to the visual version, except that the attender was blindfolded.

### The Trapdoor Card Trick, Visual Version

**Figure 2.**
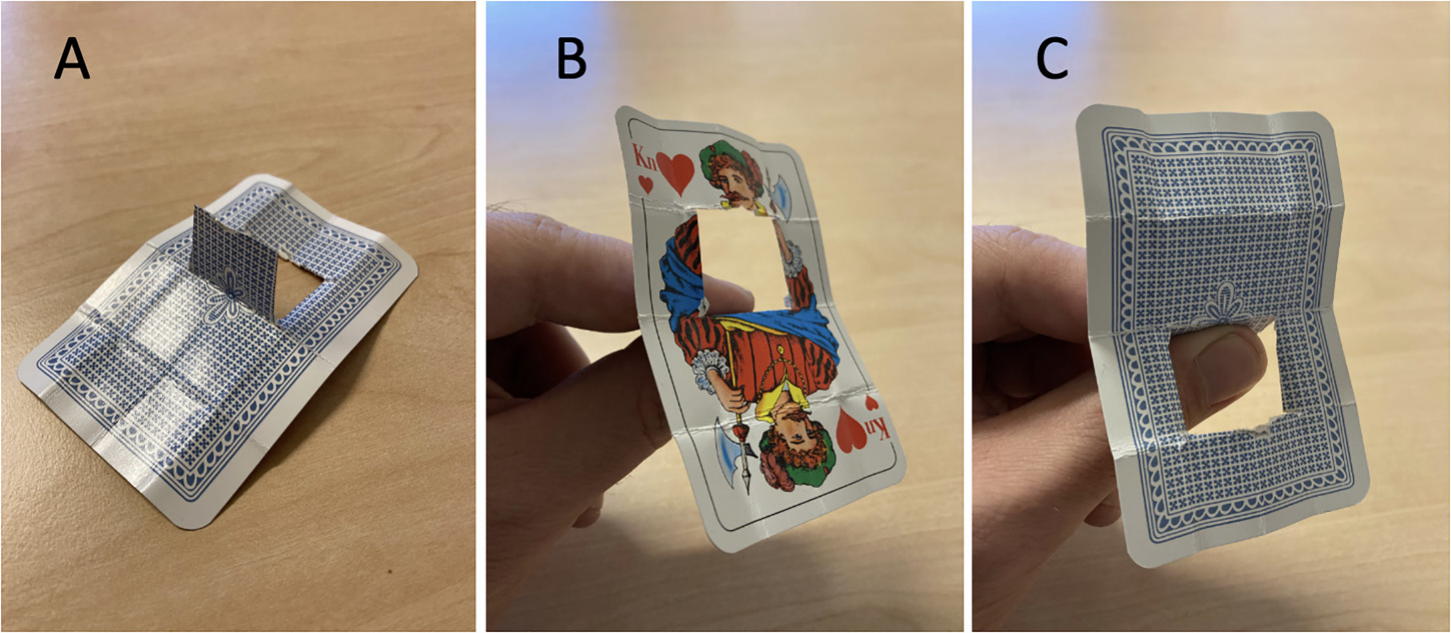
(A) The card used in the trapdoor card trick is prepared by folding it along three lines along the length of the card and three lines along the width of the card. Two of the 16 resulting “rectangles” form a “door,” which is cut out of the card along three lines. (B) At the beginning of the trick, the attender holds on to the “door,” and the face of the card faces upwards. (C) After some folding performed by the magician, while the attender holds on to the door, the back of the card faces up.

The trapdoor card trick (see Movie 4) was performed with a normal playing card with a “trapdoor” made by cutting a whole in the card, as shown in Figure 2A. The attender holds on to the “door” as shown in Figure 2B, and the magician does some folding and unfolding of the card along the pre-creased lines visible in Figure 2, leading to the situation shown in Figure 2C. Most people will probably feel that it is not possible to go from the situation in panel B to that in panel C without letting go of the “door,” although it actually is. Informal observations suggest that many people have a strong feeling of impossibility even though the folding leading from the situation in panel B to that in panel C is performed right in front of their eyes. The folding sequence leading from B to C can be seen in Movie 4. Considering that the folding is performed openly, there is no hidden secret in this trick.

### The Trapdoor Card Trick, Non-visual Version

In the non-visual version of this trick, the playing card was replaced by a piece of sandpaper of the same shape and size. Since the sandpaper was rough on one side and smooth on the other, the participants could feel which side was facing up before and after the folding. The performance was essentially identical to that of the visual version, except that the participants were encouraged to feel which side was up before and after the folding.

### The Linking Paperclips Trick, Visual Version

**Figure 3.**
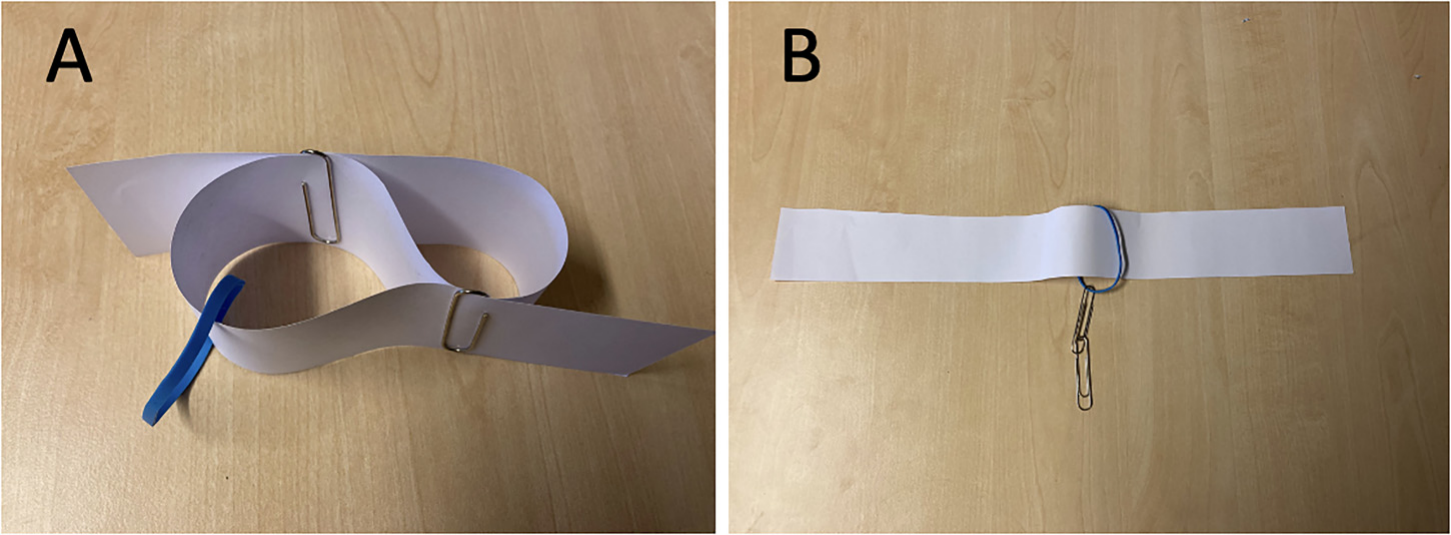
(A) The linking paperclips trick starts with a stripe of paper folded as shown in the figure, with two separate paperclips each holding two parts of the strip together and a rubber band around the paper strip in one of the loops. (B) When pulling the free ends in A apart, this situation automatically occurs, where the two paperclips are interlinked, and one of them is linked to the rubber band.

In this trick (see Movie 5), a strip of paper, two paperclips, and a rubber band were pre-arranged as shown in Figure 3A, and the attender is free to inspect the arrangement from any angle they would like. The attender is then encouraged to pull the strip straight, by pulling the two ends of the strip apart. When this is done, the two paperclips become linked to each other, and one of the paperclips become linked to the rubber band, as shown in Figure 3B. As with the trapdoor card trick, there is no hidden secret behind the trick, and although the result of pulling the strip straight is highly counterintuitive, it is simply what happens.

### The Linking Paperclips Trick, Non-visual Version

In the non-visual version of this trick, the attender was invited to explore the pre-arranged configuration (Figure 3A) using their hands and encouraged to ascertain that the two paperclips and the rubber band were separate. They were then asked to pull the strip straight by pulling the two ends of the strip apart. They were then immediately instructed not to let go of the strip, but move their hands towards the middle and then feel what objects were attached to the strip. In principle, it is possible to ask an attender to construct the initial configuration (Figure 3A) themselves, which may enhance the attenders’ appreciation of the trick, but we opted against this based on informal observations suggesting that it is often time-consuming to get the configuration right based only on verbal instruction. In order to preserve the comparability of the two versions of the tricks, we also used a pre-arranged configuration in the visual version, although it may be better for the magician to show how the arrangement is being made in that case.

## Results

### Statistical Analyses

Our main hypothesis is that the non-visual versions of the imagery tricks are roughly as good as the original visual versions. In order to assess how similar or different the different versions of the trick are likely to be in light of the data, we performed Bayesian estimation of the differences in magic ratings, as well as the differences in the probability of having ideas about the secret behind the tricks. To analyze the magic ratings, we applied ordinal regression modeling (Bürkner & Vuorre, 2019) using the **brms** package (v2.20.1, Bürkner, 2017) and R (v4.2.2, R Core Team, 2022). More specifically, we used a cumulative model with a logistic distribution function (see Bürkner & Vuorre, 2019) and default priors. In the model, we included group and trick as fixed factors (including interactions) and subject as a random factor. As pointed out by Liddell and Kruschke (2018), there are important reasons for analyzing ordinal data using ordinal models rather than the more frequently used metric models. An inconvenience of the ordinal regression model, however, is that the parameter estimates we are interested in refer to an underlying metric variable with little direct relation to the ordinal values in the data. In order to facilitate a more intuitive understanding of the parameter estimates, we followed the approach of Kruschke (2014) and also present them expressed in terms of an underlying metric variable obtained by rescaling the original one linearly such that it has a value of 0.5 at the estimated threshold between ratings of 0 versus 1 and a value of 9.5 at the estimated threshold between ratings of 9 versus 10.

**Figure 4.**
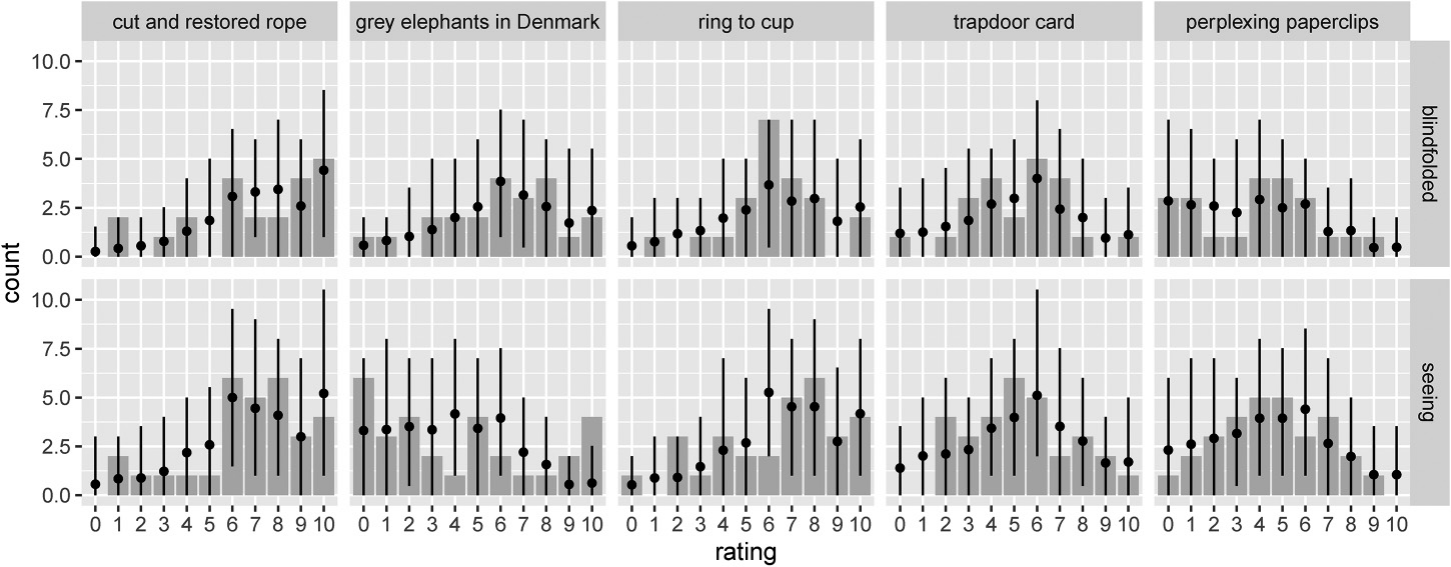
Distributions of the magic ratings for each of the five tricks, plotted separately for the two experimental groups. Results for the blindfolded group are shown in the top row, those for the seeing group in the bottom row. The gray bars show the raw data counts, while the black dots and the error bars represent the posterior predictive distribution of the cumulative ordinal regression model fitted to the data. More specifically, the dots represent the mean, while the error bars show the 2.5% and 97.5% percentiles.

**Figure 5.**
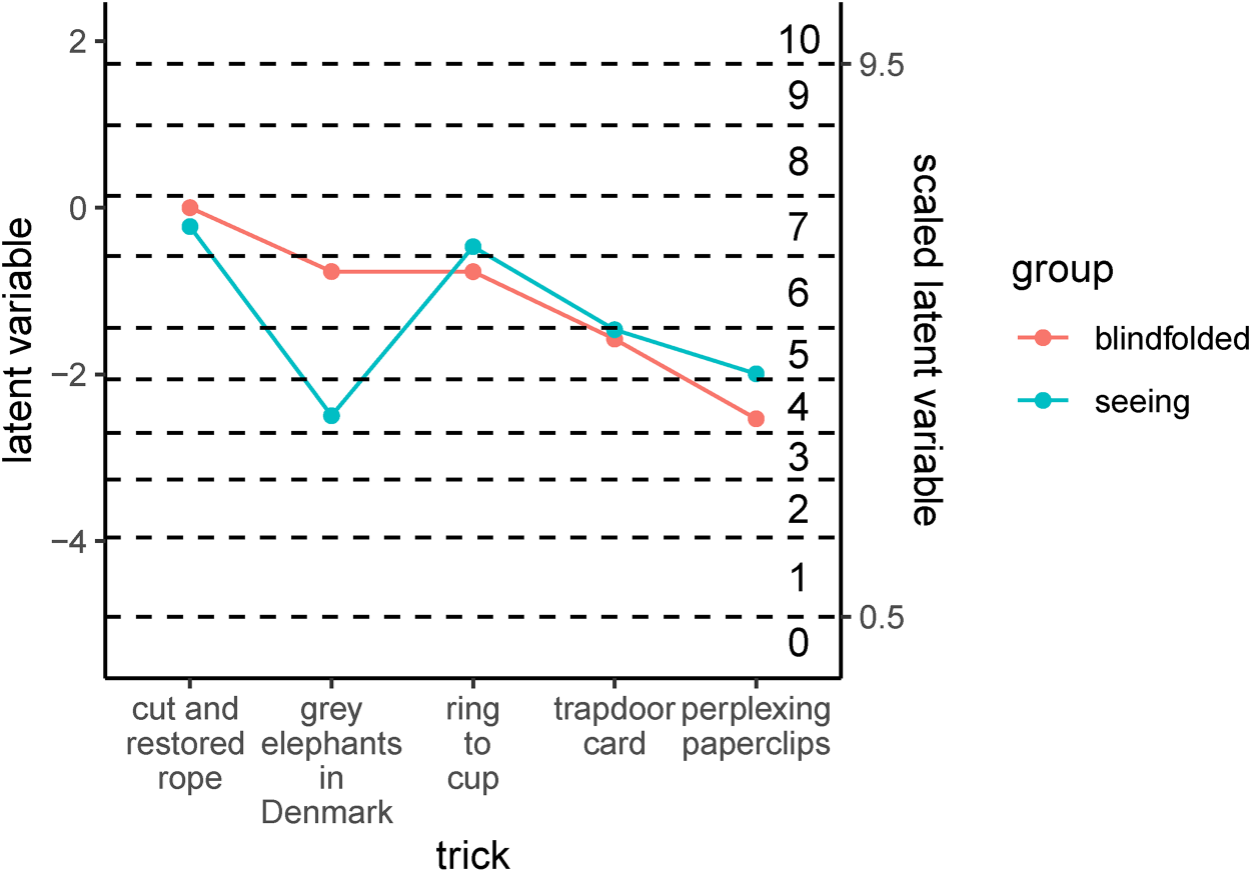
Estimated levels of magic ratings from the ordinal regression model for each trick and experimental group. The left-hand axis scale represents the original latent variable, while the scale on the right shows a linear transformation of it determined such that it has a value of 0.5 at the estimated threshold between ratings of 0 versus 1 and a value of 9.5 at the estimated threshold between ratings of 9 versus 10. The dashed lines show estimated thresholds between the ordinal categories (indicated by the numbers 1–10 shown on the left side of the right axis).

**Figure 6.**
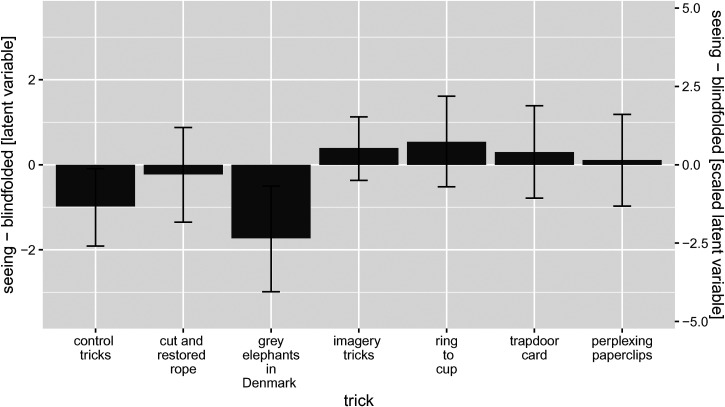
Differences between the estimates for the seeing condition and the blindfolded condition, with corresponding 95% highest density intervals (HDIs) for the posteriors. As in Figure 5, the scale on the left axis shows the value of the contrast on the original latent variable, while the scale on the right axis is linearly transformed to yield an intuitively more meaningful scale.

To analyze the participants’ answers to the questions regarding whether they had any ideas what the secret behind the various tricks might be, we computed the percentage of affirmative responses along with 95% highest density intervals (HDIs) of the posteriors for these percentages using the *binom* package (Dorai-Raj, 2014) and a uniform prior. To compute 95% HDIs for the posteriors of the *differences* between the proportions in the two experimental groups, we used the *BayesFactor* package (Morey et al., 2015). The data and R scripts for all analyses are available at https://doi.org/10.18710/ELC6OD.

**Figure 7.**
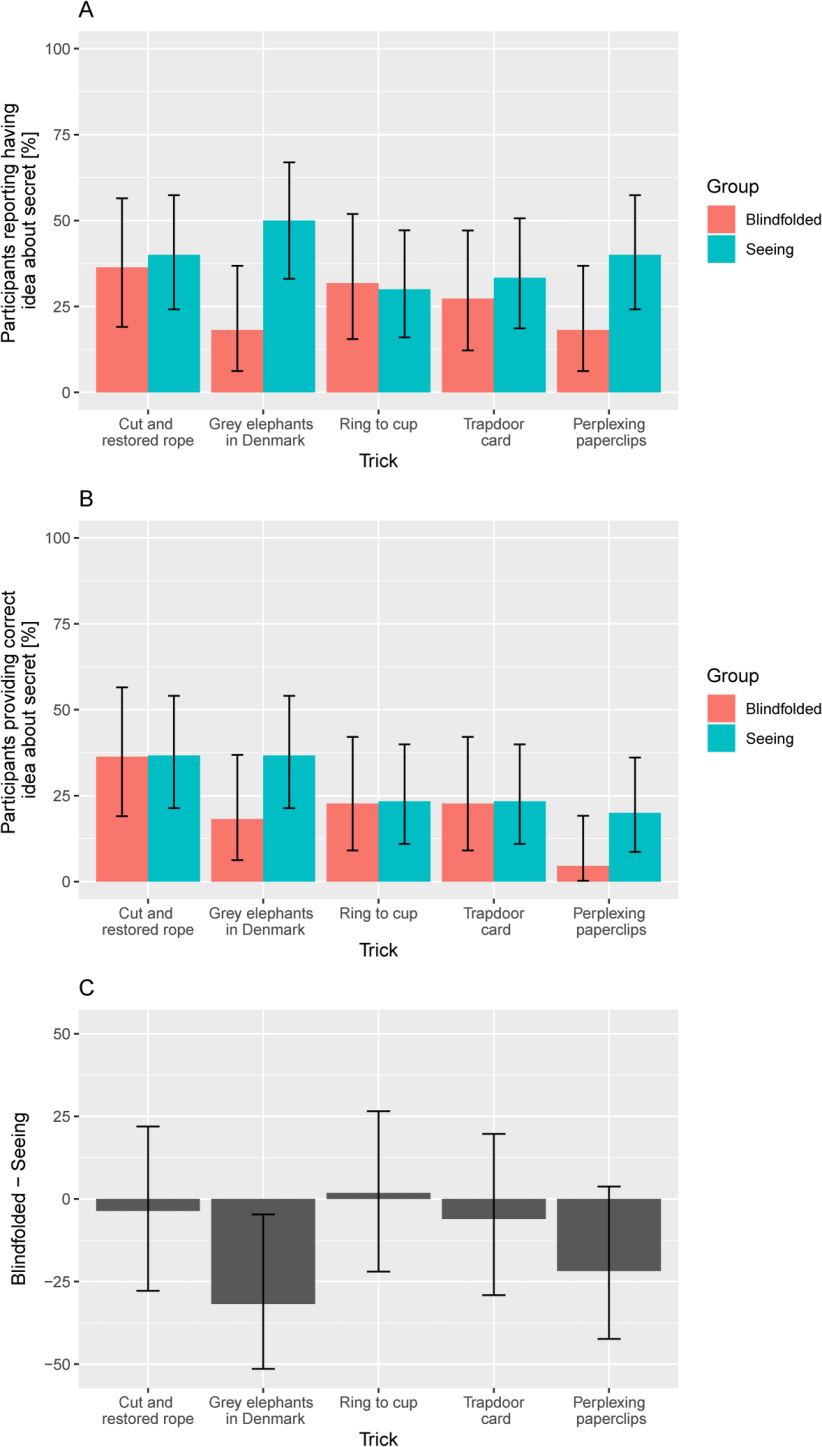
(A) Percentages of affirmative responses to the question “Do you have any idea what the secret behind this trick might be?” for the two groups of participants. The error bars show 95% HDIs for the posterior distributions based on a uniform prior. (B). Same as (A) but after categorizing positive answers as negative if the participant failed to provide any corresponding written description of his or her idea or provided an idea that was clearly implausible. (C) Differences in percentages between the two groups (as in (A)). The error bars show 95% HDIs for the posterior distributions of the differences in percentages.

### Magic Ratings

Figure 4 shows the distributions of the magic ratings for each of the five tricks, plotted separately for the two experimental groups. The gray bars show the raw data counts, while the black dots and the error bars represent the posterior predictive distribution of the cumulative ordinal regression model fitted to the data. As can be seen, the distributions are rather broad, indicating large individual variability.

**Figure 8.**
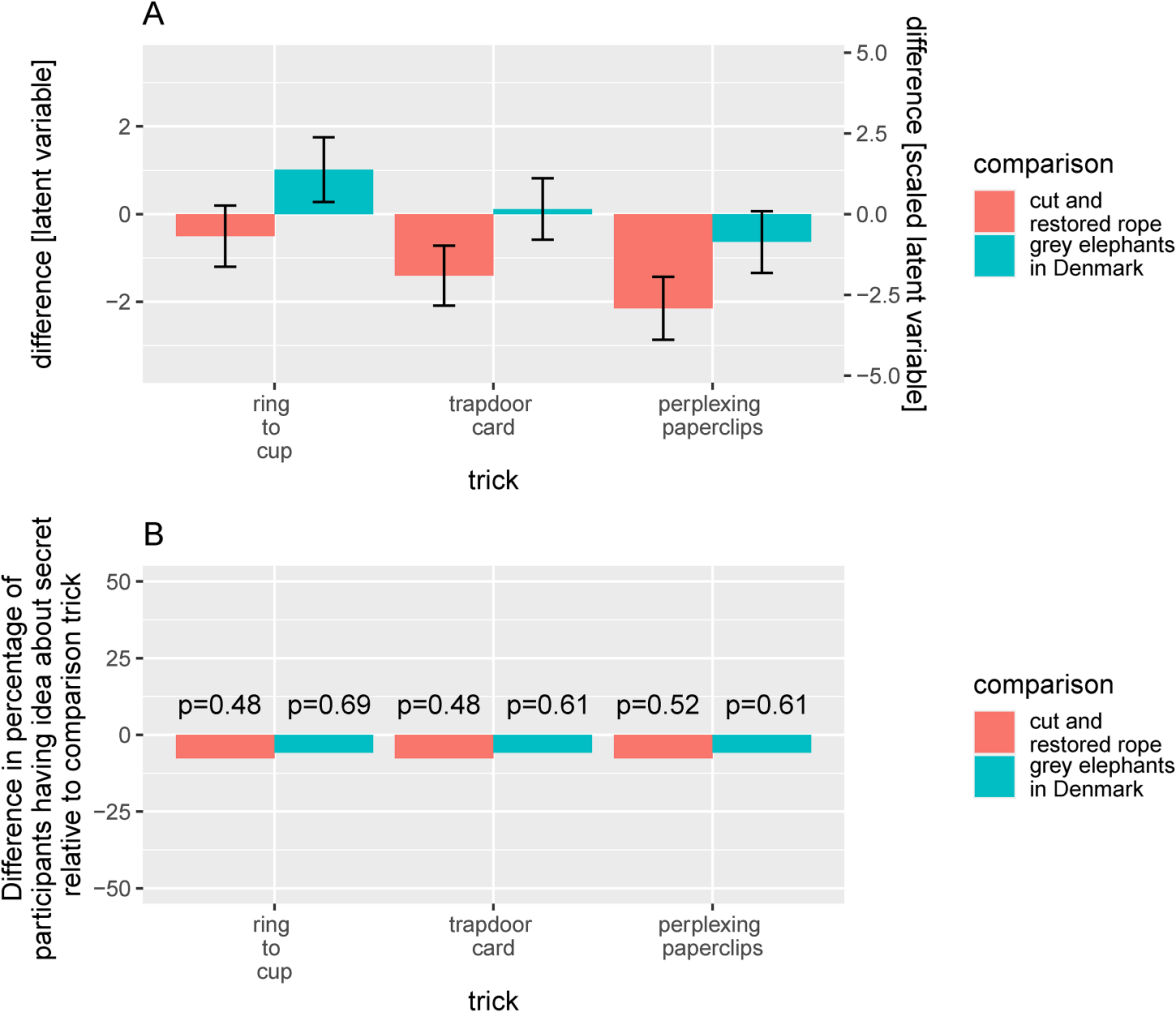
(A) Differences between the imagery tricks and each of the two control tricks with respect to the latent variable representing the level of the magic ratings. As in Figure 5, the scale on the left axis shows the value of the contrast on the original latent variable, while the scale on the right axis is linearly transformed to yield an intuitively more meaningful scale. The error bars show 95% HDIs of the posterior distributions. (B) Differences between the imagery tricks and each of the two controls tricks with respect to the percentage of participants who indicated having an idea about the secret behind the trick. The *P*-values printed in panel (B) stem from McNemar tests.

Figure 5 shows the estimated levels of magic ratings from the ordinal regression model for each trick and experimental group. The dashed lines show estimated thresholds between the ordinal categories (indicated by the numbers 1–10 shown on the left side of the right axis). For all tricks except “grey elephants in Denmark,” the scores obtained for the blindfolded group are quite similar to the ones obtained for the seeing group.

Figure 6 plots the differences between the estimates for the seeing condition and the blindfolded condition, with corresponding 95% HDIs for the posteriors. The 95% HDI of the posterior mean that, in light of the data, the probability that the true difference lies within it is 95%. Thus, the 95% HDI are useful for gaining an idea of how large the true differences may plausibly be, in light of the data. In addition to the single differences, contrasts corresponding to the same difference for (1) the average of the control tricks (cut and restored rope and grey elephant in Denmark) and (2) the average of the imagery tricks (the other three) are shown, again along with corresponding 95% HDIs for the posteriors. As in Figure 5, the scale on the left axis shows the value of the contrast on the original latent variable, while the scale on the right axis is linearly transformed to yield an intuitively more meaningful scale, as explained above.

Remember that the *cut and restored rope trick* and the *grey elephants in Denmark* trick can be considered control tricks in the sense that they were presented in the same way to both groups. Thus, it is not surprising that the difference between the two experimental groups is close to zero for the former trick, but somewhat unexpected that the difference for the latter trick is larger, and that the corresponding 95% HDI excludes zero (see Figure 6).

The three remaining tricks were performed differently in the two groups. The participants in the *seeing group* saw a standard version of the trick, while those in the *blindfolded group* experienced an adapted version of the trick which was designed to be suitable for people who cannot see. As can be seen in Figure 5 the estimated level of the ratings are similar across the versions. The levels are slightly higher for the standard versions than for the adapted versions, but the 95% HDIs for the differences (Figure 6) includes zero in all cases, so there is no strong statistical evidence for a systematic difference.

During the execution of the experiment, the experimenter noticed that the *grey elephants in Denmark trick* often failed because the participant could not think of an animal, picked another country starting with D (such as the Dominican Republic), or computed the wrong number. This happened slightly more often in the seeing condition (8 out of 30 cases, i.e., 27%) than in the blindfolded condition (5 out of 22 cases, i.e., 23%), but this cannot account for the curious difference in magic ratings for this trick, since removing these cases from the analysis only reduced the estimated difference in the level of magic ratings for the two experimental groups by 14%, and the 95% HDI still excluded zero.

### Having Ideas About the Secret Behind the Trick

Figure 7A shows the percentage of affirmative responses to the question “Do you have any idea what the secret behind this trick might be?” The error bars show 95% HDIs for the posteriors computed using the *binom* package (Dorai-Raj, 2014) and a uniform prior. Pooled across all tricks and versions, the overall proportion of affirmative responses was 33%. In each case when an affirmative response was given, the participant was asked to describe her/his idea by typing it into a corresponding text box on a computer. Qualitative analysis of these responses suggests that in the majority of the cases in which a participant had given an affirmative response to the first question, they had also provided ideas which were at least plausible or partial descriptions of the secret(s) behind the trick. In a minority of the cases, the participant failed to provide any corresponding written description of his or her idea or provided an idea that was clearly implausible. Figure 7B shows the percentage of affirmative responses which was supported by a written response that we deemed to be at least a plausible or partial descriptions of the secret(s) behind the trick. The overall proportion of responses meeting this criterion was 25%. Figure 7C shows the differences between the two groups from Figure 7A with 95% HDIs for the posteriors for those difference computed using the *BayesFactor* package (Morey et al., 2015).

As can be seen in panels A and C of Figure 7, the percentage of affirmative responses for the *cut and restored rope trick* were similar in both groups, which is not surprising considering that one and the same version of the trick was presented to both groups. The percentage of affirmative responses for the *grey elephants in Denmark trick,* however, was quite a bit higher in the *seeing group* (50%) than in the *blindfolded group* (18%), and the 95% HDI for this difference did not include zero (see Figure 7C), which was unexpected given that the trick was presented in the same way to both groups. Note that there was some indication of a corresponding difference for the grey elephants in Denmark trick in the magic ratings (see Figures 5 and 6). The percentage of affirmative responses was similar in both groups for the *ring to cup trick* and the *trapdoor card trick*. For the *linking paperclips trick*, the percentage of affirmative responses is somewhat higher in the seeing group (40%) than in the blindfolded group (18%), but the 95% HDI for the difference encloses zero.

If we remove the cases where the *grey elephants in Denmark trick* failed (see above), the difference in the percentage of affirmative responses for that trick (−31.8%) is only slightly smaller (−31.0%), with a 95% HDI spanning from −53.9% to 0.7%.

### Differences Between the Tricks

Figure 8A shows the differences between the imagery tricks and each of the two control tricks with respect to the latent variable representing the level of the magic ratings in the previously mentioned ordinal regression analysis. The error bars show 95% HDIs for these differences. At the descriptive level, all the imagery tricks are inferior to the *cut and restored rope trick*, but the difference is quite small for the *ring to cup trick*, and the 95% HDI for the difference includes zero. For the remaining two imagery tricks, the 95% HDIs exclude zero, suggesting that the differences are statistically robust. Compared to the grey elephants in Denmark trick, the ring to cup trick is superior (with a 95% HDI for the difference that excludes zero), while the trapdoor card is very similar and the perplexing paperclips trick is slightly inferior (with a 95% HDI for the difference that does not exclude zero).

Figure 8B shows the difference in the percentage of participants indicating having an idea about the secret behind the trick compared to each of the two control tricks. McNemar tests (*p*-values shown in the figure) indicate that none of the differences are statistically significant.

### Relationship Between Magic Ratings and Having Ideas About the Secret Behind the Trick

**Figure 9.**
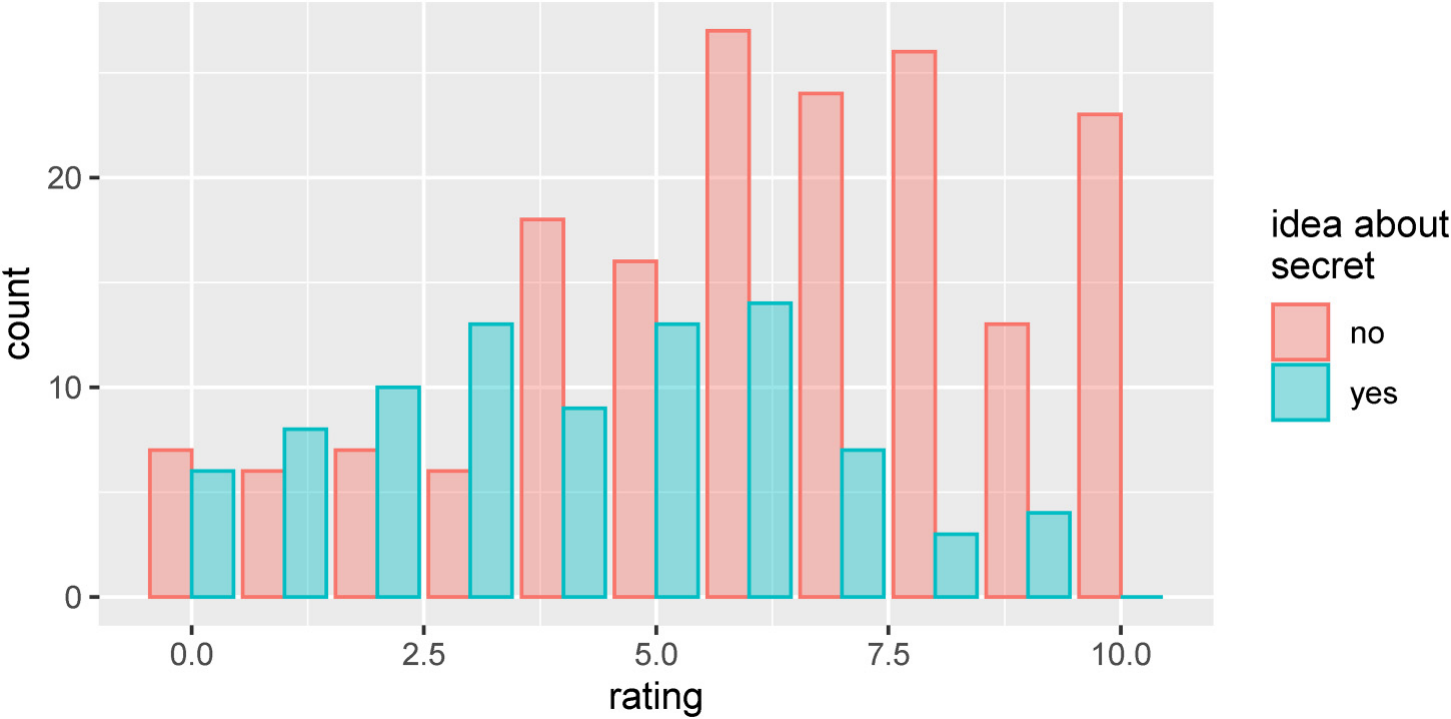
Distribution of the magic ratings plotted separately depending on whether the participant's response to the question “Do you have any idea what the secret behind this trick might be?” was affirmative or negative.

Figure 9 shows the distribution of the magic ratings plotted separately depending on whether the participant's response to the question “Do you have any idea what the secret behind this trick might be?” was affirmative or negative. As one might naively expect, the magic ratings tend to be higher in cases where a participant answered that they did not have any idea about the secret behind the trick than for cases where they did. As is readily apparent, however, the relationship between having an idea about the secret of the trick and the corresponding magic rating is rather weak. In quite a few cases where a participant stated having no idea about the secret, they nevertheless gave a low or even a zero magic rating. Conversely, in quite a few cases where a participant stated having an idea about the secret, relatively high magic ratings were given.

## Discussion

The most important result of this study is that the versions of the imagery tricks adapted for non-visual presentation were roughly comparable to the standard versions presented visually, both with respect to the participants’ magic ratings (Figure 5) and with respect to the percentage of participants indicating having an idea about the secret behind the trick (Figure 7). More specifically, we can conclude that the differences between two versions of the same trick are highly likely (posterior probability 95%) to be less than or equal to the (absolute) values at the borders of the 95% HDIs shown in Figures 6 and 7C. This result is encouraging because it suggests that adapting standard tricks based on illusions of imagery for non-visual presentation is a promising avenue for making the art of magic more accessible for people who are blind or visually impaired. We have only investigated three particular tricks and their specific adaptations for non-visual presentation, but we believe that quite many tricks are based on illusions of imagery (Ekroll, 2019) rather than visual illusions and may therefore be readily adapted for non-visual presentation.

The finding that the non-visual versions of the imagery tricks were roughly comparable to the original ones is important. Still, it is of some interest to consider how they compare to other magic tricks. When compared to the grey elephants in Denmark trick, which we included in this study as a prototypical “conjuring with information” (mental magic) trick, the level of the magic ratings for the ring to cup trick was higher, that for the trapdoor card was slightly higher, and that for the linking paperclips trick was a bit lower (see Figure 8A). Only the former difference was statistically robust. Thus, based on the magic ratings, our best estimate is that the imagery tricks tend to be better than or roughly similar to the grey elephant in Denmark trick. Based on the percentage of participants who indicated having an idea about the secret behind the trick, which was slightly less for each of the imagery trick when compared to the grey elephants in Denmark trick (Figure 8B), but not statistically significantly so according to McNemar tests, our best estimate is that the imagery tricks are slightly better than the grey elephants in Denmark trick. When compared to the cut and restored trick, which we included as a prototypical inherently visual trick, the levels of the magic ratings for the imagery tricks were all lower (Figure 8A), although the level of the magic ratings for the ring to cup trick was quite similar, with a difference of zero well within the 95% HDI (see Figure 8A). The percentage of participants who indicated having an idea about the secret behind the trick was lower for the imagery tricks than for the cut and restored rope trick (Figure 8B), which nominally suggests that the imagery tricks are better, but these differences were small and not statistically significant according to McNemar tests.

We may also compare the average magic ratings of the imagery tricks with magic ratings obtained in other studies. Beth and Ekroll (2015) reported magic ratings of videos of 13 magic tricks (see their Figures 2 and 3) performed by a semi-professional magician. The average magic rating in their study is less than 4. The average ratings for the imagery tricks in the present study are similar (*linking paperclips trick, M = 4.27*) and higher (*ring to cup trick, M = 6.35* and *trapdoor card trick, M = 5.23*) than that. Svalebjørg et al. (2020) reported magic ratings of videos of nine different tricks of three types performed by a semi-professional magician (see their Figure 3). The average rating for each of the nine different tricks ranged from 4.4 to 6.5 (see their Figure 3A). The average magic ratings of both the ring to cup trick and the trapdoor card trick investigated fall within that range, while the average magic rating of the linking paperclips trick falls somewhat below that range. Thus, it appears reasonable to conclude that the imagery tricks investigated in the present study are quite decent magic tricks.

In this study, we investigated unconnected, single tricks presented in a random sequence, and did not direct any efforts towards enhancing the participants enjoyment of the tricks via showmanship, embedding in a meaningful narrative, or other performative elements that are commonly believed to play an important role in shaping the experience of magic (e.g., Hugard & Braue, 1961; Leddington, 2020; Neale, 2015; Nelms, 2012; Stone, 2014). When using the tricks for their intended purpose, though, we believe that the use of such elements of showmanship and presentation technique can boost their effectiveness in creating experiences of magic even further.

An important issue that needs to be addressed in further work is whether the results obtained with blindfolded sighted participants in this study are transferrable to people who are blind or visually impaired. One may think of several reasons why people who are blind and people who are seeing but blindfolded may experience the tricks in different ways. Furthermore, it would be of interest to compare the experiences of people with early blindness with those people with late blindness.

An attractive feature of the imagery tricks investigated in this study is that it appears straightforward to present them to a mixed audience with one blind or visually impaired attender feeling the trick and several sighted attenders seeing the trick at the same time. In future work, it would be interesting to investigate to what extent performances of these tricks to a mixed audience can facilitate the creation of shared experiences of wonder including both blind and sighted attenders.

It seems reasonable to conclude that the imagery tricks investigated in the present study, as well as other tricks based on illusions of imagery (Ekroll, 2019) may turn out to be valuable tools for making the art of magic more accessible for people who are blind or visually impaired.

In addition to their potential applied value, these tricks are also rather interesting from a basic science perspective (Ekroll, 2019; Kuhn, 2019; Kuhn et al., 2008; Macknik et al., 2008; Thomas et al., 2015). It is probably fair to say that that currently known principles of perception and cognition do not go very far in explaining why these tricks evoke experiences of magic.

## Supplemental Material

**Figure:** Video 1.

**Figure:** Video 2.

**Figure:** Video 3.

**Figure:** Video 4.

**Figure:** Video 5.

## Notes

1. The master card mechanic and close-up magician Richard Turner is a notable exception. See Jay (2021).

2. For lack of a better term, we use this one to denote “spectators” or “an audience” in a broader sense not etymologically associated with particular senses.

3. We borrow the term *conjuring with information* from Teller, who used it in his introduction to Banachek's (1998) book.

4. Some people may also think of ostriches in the Domenican Republic, or elephants in Germany in a German-speaking context, because the German name of Germany is “Deutschland.”
